# Analysis of the population-level impact of co-administering ivermectin with albendazole or mebendazole for the control and elimination of *Trichuris trichiura*

**DOI:** 10.1016/j.parepi.2016.02.004

**Published:** 2016-03-02

**Authors:** Hugo C. Turner, James E. Truscott, Alison A. Bettis, T. Déirdre Hollingsworth, Simon J. Brooker, Roy M. Anderson

**Affiliations:** a London Centre for Neglected Tropical Disease Research, Department of Infectious Disease Epidemiology, School of Public Health, Faculty of Medicine, St Mary's Campus, Imperial College London, Norfolk Place, London W2 1PG, UK; b Department of Infectious Disease Epidemiology, School of Public Health, Faculty of Medicine, St Mary's Campus, Imperial College London, Norfolk Place, London W2 1PG, UK; c Mathematics Institute, University of Warwick, Coventry CV4 7AL, UK; d School of Life Sciences, University of Warwick, Coventry CV4 7AL, UK; e Faculty of Infectious and Tropical Diseases, London School of Hygiene & Tropical Medicine, London, UK

**Keywords:** Soil-transmitted helminth, *Trichuris trichiura*, Ivermectin co-administration, Elimination, Control, Mass drug administration, ALB, albendazole, R_0_, basic reproductive number, ERRs, egg reduction rates, IVM, ivermectin, MBZ, mebendazole, Pre-SAC, preschool-aged, SAC, school-aged children, STH, soil-transmitted helminth, WASH, water, sanitation and hygiene, WHO, World Health Organisation

## Abstract

**Introduction:**

Soil-transmitted helminth (STH) infections are predominately controlled by providing children with preventive chemotherapy with either albendazole or mebendazole. However, neither has a high efficacy against *Trichuris trichiura*. This low efficacy limits the overall effectiveness of the current STH control programmes against *T. trichiura*. It has been demonstrated that co-administering ivermectin with albendazole or mebendazole significantly increases the efficacy of current treatments, which may increase the overall effectiveness of control programmes.

**Methods:**

Using a STH transmission mathematical model, we evaluated the potential impact of co-administering ivermectin with albendazole or mebendazole to treat *T. trichiura* within a preventive chemotherapy programme targeting children (2–15 year olds). We evaluated the impact in terms of reduction in prevalent infections, mean worm burden, and prevalence of heavy infections.

**Results:**

Although the current treatment strategy reduced *T. trichiura* worm burden and prevalence of heavy infections, due to their poor efficacy the long term impact of preventive chemotherapy for children was smaller compared to the other STH. Co-administering ivermectin increased the projected impact of the preventive chemotherapy programme in terms of all three of the explored metrics, practically in high transmission settings. Furthermore, ivermectin co-administration greatly increased the feasibility of and timeframe for breaking transmission.

**Conclusions:**

Co-administering ivermectin notably increased the projected impact of preventive chemotherapy in high transmission settings and increased the feasibility for breaking transmission. This has important implications for control programmes, some of which may be shifting focus from morbidity control to interruption of transmission, and some of which may be logistically unable to provide preventive chemotherapy twice a year as recommended. However, the benefit of co-administering ivermectin is limited by the fact that 2–5 year olds are often ineligible to receive treatment.

## Highlights

• The impact of chemotherapy against *Trichuris* is smaller compared to what can be seen for the other soil-transmitted helminths.
• Co-administering ivermectin increases the projected impact of preventive chemotherapy.
• It also has the potential to interrupt transmission in some settings.

## 1 Introduction

The most common neglected tropical diseases (NTDs) are the soil-transmitted helminths (STH) which include *Ascaris lumbricoides*, *Trichuris trichiura* and the hookworms (Pullan and Brooker, 2012). The mainstay of the control of STH is regular preventive chemotherapy using either albendazole or mebendazole. Control programmes largely target these treatments at school-aged children (SAC) and preschool-aged children (Pre-SAC). In the majority of endemic areas, treatment is given annually, but in areas of intense transmission (defined as a prevalence of any STH greater than 50% in SAC), the WHO recommends that the treatment frequency is increased to at least twice a year (depending on resource availability) (World Health Organization, 2006). The aim of these WHO guidelines is to reduce the prevalence of heavy infections and their associated morbidity (World Health Organization, 2012).

However, although both albendazole and mebendazole have a high efficacy against *A. lumbricoides* (with cure rates above 78%), mebendazole does not effectively clear hookworm infections, and neither drug has an adequate efficacy against *T. trichiura (*Keiser and Utzinger, 2008, Vercruysse et al., 2011a, Levecke et al., 2014a) (albendazole has a cure rate of 28% (95% CI: 13%–39%) and mebendazole 36% (95% CI: 16%–51%) (Keiser and Utzinger, 2008)). This considerably lower efficacy of the standard treatments limits the overall possible effectiveness of preventive chemotherapy against *T. trichiura*, which is estimated to infect 464.6 million people worldwide (mainly in sub-Saharan Africa and Asia but also in parts of Latin America (Pullan et al., 2014a)). Heavy *T. trichiura* infections can lead to severe anaemia, growth retardation, and impaired cognitive development (Bundy and Cooper, 1989). In addition to these clinical and nutritional impacts, *T. trichiura* may have effects on the physical fitness of children (Yap et al., 2014).

In addition to the goals of reducing morbidity, there is growing interest in investigating the feasibility of interrupting transmission of STH (Bill & Melinda Gates Foundation, 2014, Brooker et al., 2015, Anderson et al., 2015) — although this is not yet the aim of current World Health Organisation (WHO) policy. In this regard, the poor treatment efficacy of albendazole and mebendazole against *T. trichiura* may hinder the success of STH elimination programmes as a whole — as residual *T. trichiura* infection may remain after the transmission of *A. lumbricoides* and hookworm is interrupted.

The impact of a STH control programme on *T. trichiura* may be enhanced by adding ivermectin to the treatment schedule. This approach has been demonstrated to significantly increase the treatment efficacy of both standalone albendazole and mebendazole against *T. trichiura* (Knopp et al., 2010, Beach et al., 1999, Belizario, 2003, Speich et al., 2015). Ivermectin is an anthelmintic which Merck & Co donate for the control of onchocerciasis and lymphatic filariasis (25 Years: The MECTIZANⓇ Donation Program).

In this paper we use mathematical models of STH transmission (Truscott et al., 2014, Truscott et al., 2015, Anderson et al., 2014, Turner et al., 2016) to evaluate the following factors; 1) the additional programmatic impact gained by co-administering ivermectin in terms of reducing mean worm burden, prevalent infections, and the prevalence of heavy infections; and 2) how ivermectin co-administration may influence the feasibility of interrupting the transmission of *T. trichiura*.

## 2 Methods

This analysis was performed using a fully age-structured deterministic model of the dynamics of STH transmission including simulation of parasite sexual reproduction (Truscott et al., 2014, Truscott et al., 2015, Anderson et al., 2014, Turner et al., 2016). In this paper we used the model parameters pertaining to *T. trichiura* which are described in more detail in the Supporting Table S1. It should be noted that the individuals treated each round are effectively chosen at random from the relevant age group(s) and the model does not currently address systematic non-compliance. A future publication will examine this issue in more detail using an individual-based stochastic model.

### 2.1 Fitted setting

**Fig. 1:**
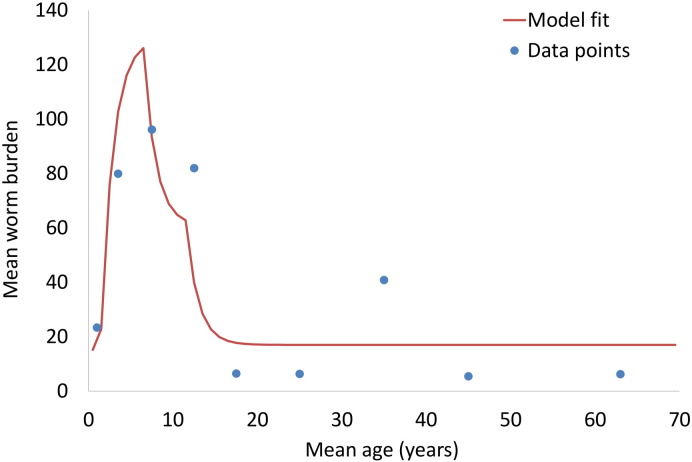
Model fit to cross-sectional data from the St. Lucia study (Bundy et al., 1987). Note that the model fit was done to the individual data points and not the average data values shown here (see Supporting Information and Supporting Fig. 1).

The model parameters describing the age-specific exposure and contribution to the infection reservoir and level of transmission (R_0_) were estimated by fitting to data from the cross-sectional study of *T. trichiura* conducted in St. Lucia by Bundy et al. (1987) (Fig. 1). The study collected pre-treatment and post-treatment stool samples from 119 individuals across a full range of ages. The resulting data set contains a record for each individual consisting of age, eggs per gramme (EPG) of faeces and expelled worm burden. We estimated that in this setting, the overall R_0_ for the whole community was 1.75 (reported R_0_ estimates for *T. trichiura* often relate to the R_0_ for children only and are therefore higher).

The age profile of infection intensity (Fig. 1) is very consistent to that reported by a recent systematic review of epidemiological surveys of STH in Southeast Asia (Dunn et al., 2016).

### 2.2 Treatment efficacy

The model describes changes in the mean worm burden over time and age. Treatment impact within the model is defined in terms of the proportion of worms expelled after treatment. The efficacy values used (see below) are based on observed faecal egg reduction rates (ERRs) and not cure rates (the percentage of individuals who became helminth egg negative following treatment with an anthelmintic drug). The ERR of standalone albendazole (400 mg) was assumed to be 50% (Vercruysse et al., 2011a) and the ERR of albendazole–ivermectin co-administration was assumed to be 95%, based on a recent randomized controlled trial by Speich et al. (2015). This is consistent with a different trial which reported a similar ERR of 91% (Knopp et al., 2010). The impact of using mebendazole–ivermectin combination therapy was also considered within the sensitivity analysis (Knopp et al., 2010). Within this scenario, the ERR of standalone mebendazole (500 mg) was assumed to be 63% (Levecke et al., 2014a) and mebendazole–ivermectin co-administration to be 97%, based on the trial by Knopp et al. (2010).

Children under 15 kg in weight or 90 cm in height (generally under five years of age (Goldman et al., 2007)) are ineligible to receive ivermectin (World Health Organization, 2006) and were assumed to have only received albendazole or mebendazole. Individuals under one years of age were assumed to be ineligible for mebendazole treatment and individuals under two years of age ineligible for albendazole (400 mg) treatment. Though one year olds are sometimes provided a lower dose of albendazole (200 mg) (World Health Organization, 2006), this was not included in the model as the precise efficacy of this lower dose is unknown.

### 2.3 Programmatic scenarios and output

The model was used to simulate a programme targeting Pre-SAC and SAC, following WHO guidelines (World Health Organization 2012), and to compare standalone treatment to ivermectin co-administration using three different effectiveness metrics (described in Turner et al.'s (Epub ahead of print) work): 1) the total number of worm years averted (i.e. a year lived with a worm), 2) the total number of prevalent infection case years averted (i.e. the number of years lived with a prevalent infection prevented — not adjusted for diagnostic sensitivity), and 3) total number of heavy infection case years averted (i.e. the number of years lived with heavy infection prevented). Heavy infection was defined as having a worm burden above the age-specific thresholds for disease described in Table 1 (as defined in Chan et al.'s (1994) work and the 2010 Global Burden of Disease study (Pullan et al., 2014a)). It is important to note that the relationships between STH infection burdens (present and/or past) and morbidity are complex and not well understood (Brooker, 2010). We employed published estimates as detailed in Table 1.

**Table 1:** Age-specific worm burden thresholds for heavy infections (a proxy for morbidity) (Chan et al., 1994, Bundy et al., 2004).

Age class (years)	Number of worms — lower threshold	Number of worms — higher threshold
0–4	90	250
5–9	130	375
10 +	180	500

The lower thresholds are based on empirical observations of worm numbers associated with developmental deficits (Chan et al., 1994). The higher thresholds are more conservative values intended to reflect more serious clinical consequences and to provide a lower boundary to the estimate of morbidity (Chan et al., 1994, Bundy et al., 2004).

In addition to the fitted transmission setting (R_0_ = 1.75), we also explored a setting with lower transmission (R_0_ = 1.25). These stratifications for transmission settings differ somewhat from the WHO prevalence categories for reasons described in Truscott et al.'s (2014) work. These R_0_'s are different to previous studies on hookworm and *A. lumbricoides* (Truscott et al., 2014, Truscott et al., 2015, Anderson et al., 2014, Turner et al., 2016) due to the difference in the level of density dependence acting on female worm fecundity (Supporting Table S1). The sensitivity of the relative impact of ivermectin co-administration to different treatment coverage levels was also explored.

In addition to the increased programmatic impact regarding control of *T. trichiura*, the model was used to compare the feasibility of breaking transmission for each of the different treatment regimens, as well as the timespans required to do so. Breaking transmission is theoretically possible when worm levels are driven low enough that the probability of a female worm not encountering a mate in its host crosses a critical value. Below this, fertile egg production falls so low that the parasite population as a whole cannot be sustained (the so called ‘breakpoint’, Anderson and May, 1985).

## 3 Results

### 3.1 Controlling morbidity

**Fig. 2:**
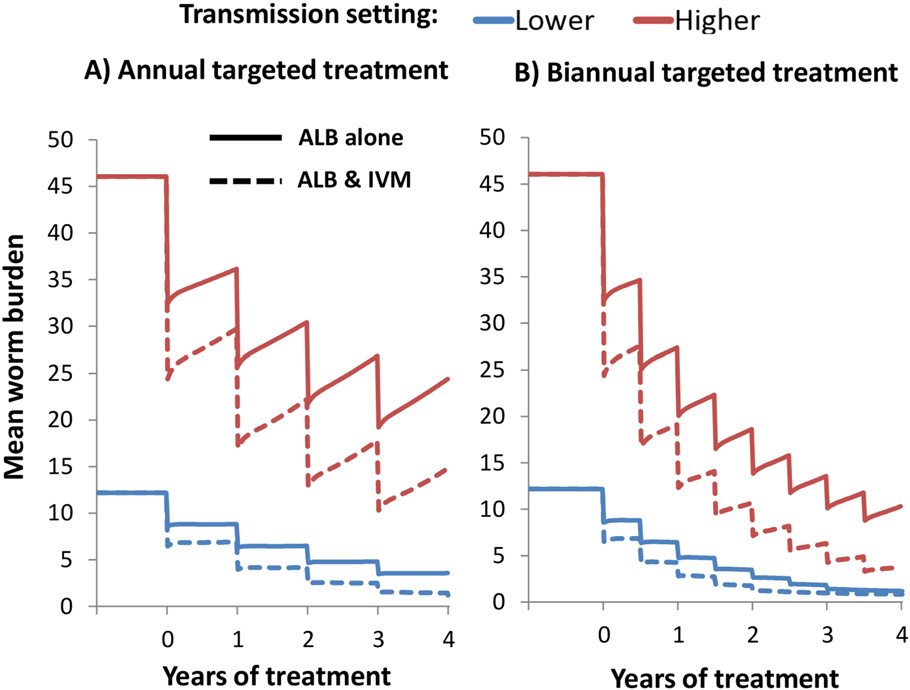
Projected impact of annual and biannual targeted preventive chemotherapy with and without ivermectin co-administration on the mean worm burden of *T. trichiura*. The solid and dotted line pertain to standalone albendazole, and albendazole–ivermectin co-administration respectively. Two different transmission settings were explored; lower (R_0_ = 1.25), and higher (R_0_ = 1.75 — fitted). Results assume 75% treatment coverage of Pre-SAC and SAC. The drug efficacy was assumed to be 50% for standalone albendazole (Vercruysse et al., 2011a), and 95% when co-administering ivermectin (Speich et al., 2015). Note that those under five years of age cannot receive ivermectin and would only be treated with albendazole. ALB; Albendazole, IVM; Ivermectin.

When using standalone albendazole (monotherapy), annual targeted PC (Pre-SAC and SAC) elicited only a gradual reduction in the overall mean worm burden of *T. trichiura* (Fig. 2).

**Fig. 3:**
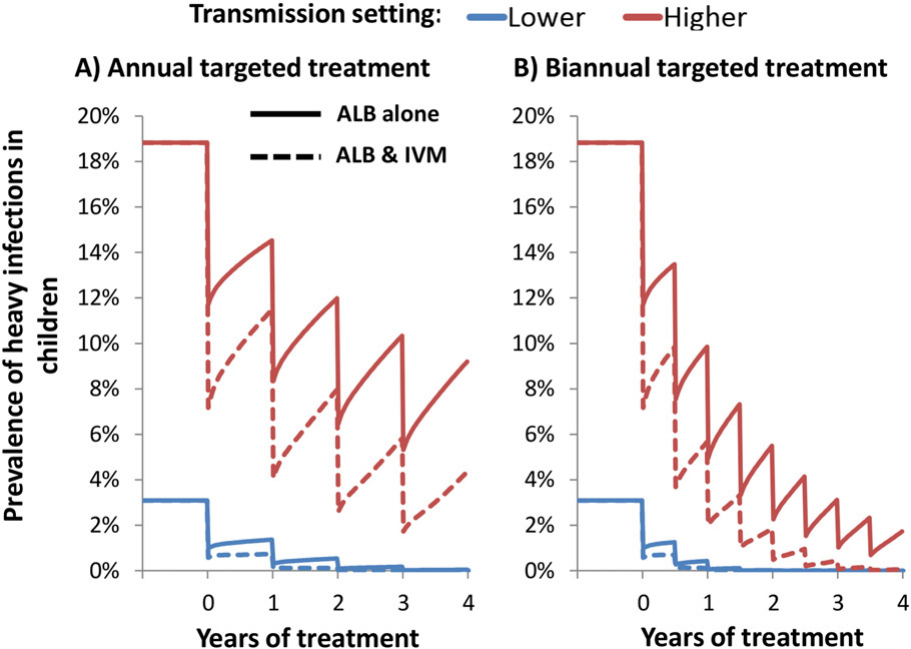
Projected impact of annual and biannual targeted preventive chemotherapy with and without ivermectin co-administration on the prevalence of heavy *T. trichiura* infections in children. The solid and dotted line pertain to standalone albendazole, and albendazole–ivermectin co-administration respectively. Two different transmission settings were explored; lower (R_0_ = 1.25), and higher (R_0_ = 1.75 — fitted). Results assume 75% treatment coverage of Pre-SAC and SAC. The drug efficacy was assumed to be 50% for standalone albendazole (Vercruysse et al., 2011a), and 95% when co-administering ivermectin (Speich et al., 2015). Note that those under five years of age cannot receive ivermectin and would only be treated with albendazole. The results assume the lower intensity thresholds for heavy infection (presented in Table 1). The corresponding results using the higher intensity threshold are presented in Supporting Fig. S2. ALB; Albendazole, IVM; Ivermectin.

In lower transmission settings annual albendazole monotherapy at a high coverage (75%) was projected to be sufficient in controlling heavy infections in children (Fig. 3 and Supporting Fig. S2). However, these simulations indicate that in the fitted (higher) transmission settings annual albendazole monotherapy may be insufficient to fully control the prevalence of heavy infections (Fig. 3 and Supporting Fig. S2). The impact of albendazole monotherapy on heavy infections was greater, the higher the intensity threshold for heavy infection (Fig. 3 and Supporting Fig. S2).

Ivermectin co-administration increased the projected impact of the programme in terms of all three of the explored metrics; worm burden, prevalent case years, and heavy infection case years, especially in high transmission settings (Table 2). Of particular note, in a high transmission setting co-administering ivermectin increased the number of heavy infection case years averted by 36% (when using the lower intensity threshold (Table 1)). Although, some heavy infections may remain if treatment is only given annually (Fig. 3).

**Table 2:** Programmatic impact in terms of the worm burden reduced, prevalent case years averted, and heavy infection case years averted, when treating with and without ivermectin co-administration.

Treatment strategy	Worm years averted (per 100)	Prevalent case years averted (per 100)	Heavy infection case years averted — lower threshold^a^ (per 100)	Heavy infection case years averted — higher threshold^a^ (per 100)
*Fitted (higher) transmission setting*				
Standalone albendazole				
Annual targeted treatment	24,290	52	55	19
Biannual targeted treatment	35,733	158	78	22
Ivermectin co-administration				
Annual targeted treatment	33,653	118	75	22
Biannual targeted treatment	40,981	390	84	23

*Lower transmission setting*				
Standalone albendazole				
Annual targeted treatment	8,995	218	12.888	0.512
Biannual targeted treatment	10,055	276	13.353	0.517
Ivermectin co-administration				
Annual targeted treatment	10,005	275	13.355	0.517
Biannual targeted treatment	10,858	363	13.538	0.518

Results assume 75% treatment coverage of Pre-SAC and SAC. Two different transmission settings were explored; lower (R_0_ = 1.25), and higher (R_0_ = 1.75 — fitted). The analysis was performed with a ten year time horizon (comparing ten years of standalone treatment to ivermectin co-administration). Note that those under five years of age did not receive ivermectin and would only be treated with albendazole.

a The thresholds for heavy infection are presented in Table 1.

**Fig. 4:**
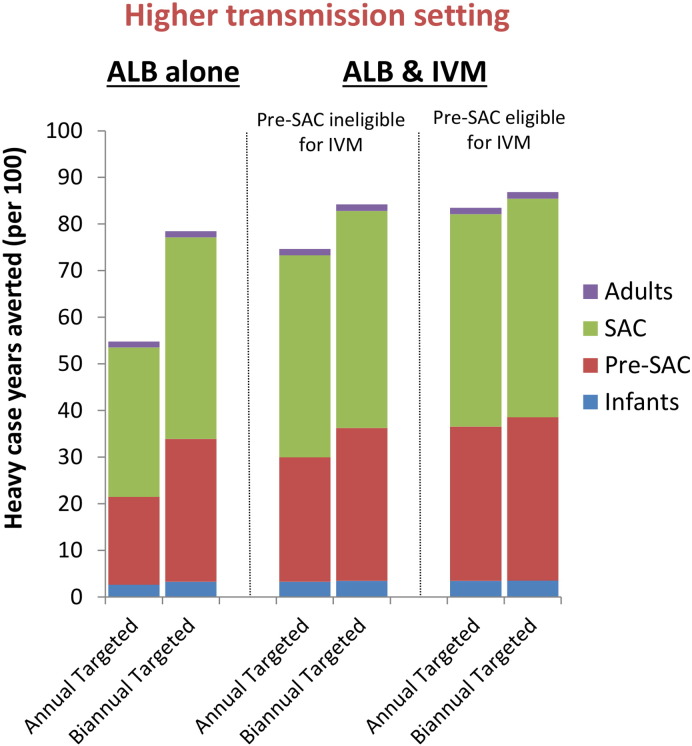
Impact of a child-targeted preventive chemotherapy in terms of heavy case years averted with and without ivermectin co-administration. The bars are stratified by the host age-group (from the bottom up: Infants (0–2 year olds), Pre-SAC (2–5 year olds), SAC (5–15 year olds), and Adults (≥ 15 year olds)). Results pertain to the fitted (higher) transmission setting (R_0_ = 1.75 — fitted) and assume 75% treatment coverage of Pre-SAC and SAC. The analysis was performed with a ten year time horizon (comparing ten years of standalone treatment to ivermectin co-administration). The drug efficacy was assumed to be 50% for standalone albendazole (Vercruysse et al., 2011a), and 95% when co-administering ivermectin (Speich et al., 2015). The results assume the lower intensity thresholds for heavy infection (presented in Table 1). The corresponding results using the higher intensity threshold are presented in Supporting Fig. S3. ALB; Albendazole, IVM; Ivermectin.

If ivermectin (or another alternative treatment) could be given to Pre-SAC, the relative impact on heavy infections averted in a high transmission setting would further increase from 36% to 52% (Fig. 4 and Supporting Fig. S3) — when using the lower intensity threshold (Table 1).

In both explored transmission settings, the impact to annual ivermectin co-administration was comparable to that of increasing the treatment frequency of albendazole monotherapy to twice a year (Table 2). Biannual treatment is recommended by the WHO in high risk settings, if the resources are available — which is not always the case (World Health Organization, 2006). Even with biannual standalone treatment at a high coverage some heavy infections were projected to remain (Fig. 3). Consequently, even if biannual treatment were feasible, co-administering ivermectin (for both rounds) would still be beneficial in some settings (Fig. 3 and Table 2).

The assumed level of treatment coverage did not greatly influence the relative increase in effects gained by co-administering ivermectin (Supporting Table S2).

The number of worm years and heavy infection case years averted due to preventive chemotherapy was higher in the higher transmission setting (Table 2). In contrast, the number of prevalent case years averted was often larger in the lower transmission setting (Table 2). This occurred because of the highly nonlinear relationship between mean worm burden and prevalence (a large proportion of worms are in a small proportion of individuals) (Turner et al., Epub ahead of print). Consequently, at lower worm burdens, changes in mean worm load will often result in larger changes in prevalence (hence the number of prevalent case years averted can decrease as the level of transmission increases).

The relative increase in benefits yielded by ivermectin co-administration increased with the level of transmission intensity (Table 3). In lower transmission settings the relative benefit in terms of averting heavy infections (Table 3) was highly dependent on the assumed intensity threshold (Table 1) i.e. the higher the intensity threshold, the easier it is to control heavy infections, and the lower the projected benefit of co-administering ivermectin. When using the higher intensity threshold, the pre-control prevalence of heavy infections was almost negligible for this transmission setting (hence there was a low benefit of ivermectin co-administration) (Supporting Fig. S2).

**Table 3:** Sensitivity of the relative increase in effects gained by co-administering ivermectin with albendazole to the transmission setting (in comparison with targeted albendazole monotherapy).

Transmission intensity	Worm years averted	Prevalent case years averted	Heavy infection case years averted — lower threshold^a^	Heavy infection case years averted — higher threshold^a^
Higher	39%	126%	36%	15%
Lower	11%	26%	4%	1%

Results pertain to a targeted preventive chemotherapy programme treating Pre-SAC and SAC with 75% treatment coverage. Two different transmission settings were explored; lower (R_0_ = 1.25), and higher (R_0_ = 1.75 — fitted). The analysis was performed with a ten year time horizon (i.e. comparing ten years of standalone treatment to ivermectin co-administration). Note that those under five years of age did not receive ivermectin and would only be treated with albendazole.

a The thresholds for heavy infection are presented in Table 1.

### 3.2 Breaking transmission

**Fig. 5:**
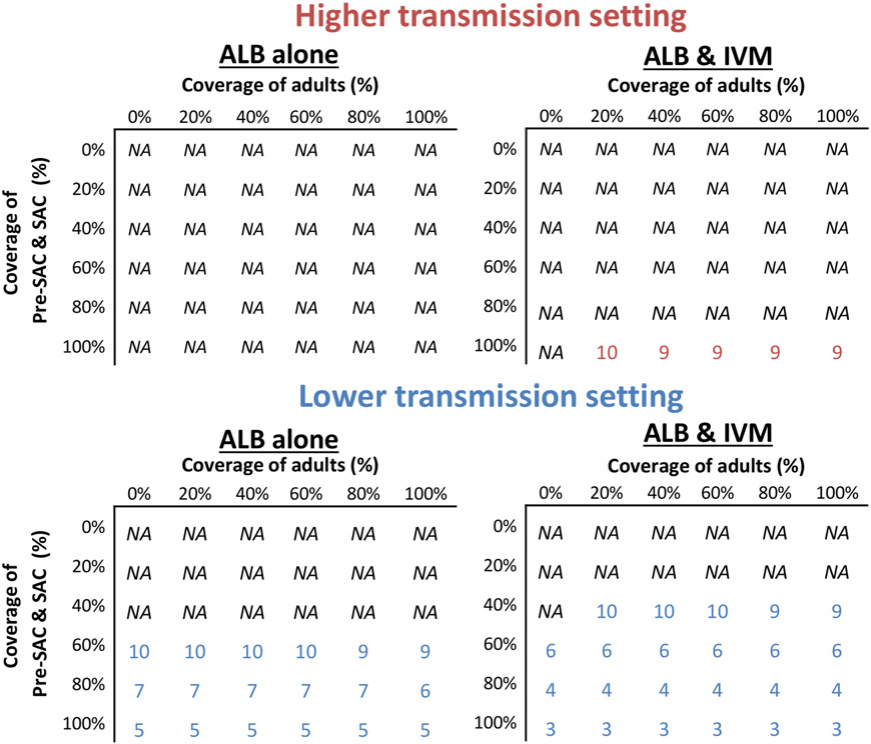
Number of years of annual treatment to achieve elimination of *T. trichiura* as a function of coverage of children versus adults. Two different transmission settings were explored; lower (R_0_ = 1.25), and higher (R_0_ = 1.75 — fitted). Note that those under five years of age did not receive ivermectin and would only be treated with albendazole. ALB; Albendazole, IVM; Ivermectin. The corresponding results for mebendazole as shown in Supporting Fig. S4. Durations over ten years were not considered (marked as NA (not achievable)).

When distributing albendazole monotherapy, breaking transmission even in the lower transmission setting was very difficult without a very high sustained coverage of Pre-SAC and SAC (Fig. 5).

Ivermectin co-administration increased the feasibility of, and decreased the length of time required for, elimination (Fig. 5). As preventive chemotherapy coverage levels increased, feasibility of elimination improved and was possible in a shorter timeframe. The projections indicate that expanding treatment to include adults would only very slightly accelerate progress to elimination (by a maximum of two years). It should be noted that, even with ivermectin co-administration, elimination was often not possible within this timeframe in settings with high transmission.

### 3.3 Mebendazole–ivermectin co-administration

A standalone treatment of mebendazole was assumed to have slightly higher efficacy compared to standalone albendazole (Vercruysse et al., 2011a, Levecke et al., 2014a), and therefore it was projected to have a slighter larger impact (Fig. 5 and Supporting Fig. S4). However, ivermectin co-administration still notably increased the programmatic effectiveness of a mebendazole-based programme and the general trends remained the same (Supporting Fig. S4). This was found to be particularly important in the context of achieving elimination — as standalone mebendazole is still insufficient to break transmission in many settings, despite its higher efficacy (Supporting Fig. S4).

## 4 Discussion

### 4.1 Controlling morbidity

Though targeting children for preventive chemotherapy with the current monotherapy treatments reduces *T. trichiura* worm burdens, the impact on *T. trichiura* was lower than on *A. lumbricoides* (despite their similar age intensity profiles) (Anderson et al., 2014, Anderson et al., 2015, Truscott et al., 2014, Truscott et al., 2015).

In lower transmission settings and at a high coverage of Pre-SAC and SAC, monotherapy was projected to be effective in controlling heavy infections in children (Fig. 3 and Supporting Fig. S2) — though moderate intensity infections may still remain and have an impact on health (Fig. 1). However, these simulations indicate that in high transmission settings annual monotherapy may be insufficient to fully control heavy infections. Co-administering ivermectin increased the projected impact of the programme, particularly in high transmission settings (though some heavy infections remained when treatment was given annually).

Increasing the treatment frequency of albendazole monotherapy to twice a year was projected to have an equivalent impact to that of annual ivermectin co-administration. Biannual treatment is recommended by the WHO in high risk settings, if the resources are available — which is not always the case (World Health Organization, 2006). However, it is also important to recognise that increasing the treatment frequency of preventive chemotherapy results in a notable increase in programmatic costs and the resources needed for delivery (Turner et al., 2013, Turner et al., 2015a). Furthermore, biannual standalone treatment did not clear all heavy infections (Fig. 3). Consequently, if biannual treatment were feasible in a certain setting, co-administering ivermectin (for both rounds) would result in additional benefit (Fig. 3 and Table 2).

These findings were slightly more striking for programmes distributing albendazole than for mebendazole (as albendazole was assumed to have a slightly lower efficacy), but the general conclusions apply to both monotherapy treatments.

### 4.2 Breaking transmission

When using the current standalone treatments, elimination of *T. trichiura* was projected to be infeasible many settings (Fig. 5). This has particularly important consequences if STH control programmes shift their goals from control of morbidity to the interruption of transmission, as it indicates that without a change in approach, many endemic areas will be unable to meet new targets and treatment will have to be continued (which has important economic implications). Ivermectin co-administration greatly increased feasibility and lowered the timeframe of repeated preventive chemotherapy required for elimination (Fig. 5) — which could potentially generate programmatic cost savings (Turner et al., Epub ahead of print).

The coverage level of Pre-SAC and SAC was the most influential on feasibility of elimination. The projections indicate expanding treatment to adults through community-wide treatment would only very slightly accelerate progress to elimination in this setting (Fig. 5). The benefit of treating adults for *T. trichiura* was less than that estimated for *A. lumbricoides*, and much less than for hookworm, due to the assumed age profile of infection intensity (derived from a set of field studies in St Lucia (Bundy et al., 1987)) — which determines the proportion of the total worm population in adults (Truscott et al., 2014, Anderson et al., 2014, Turner et al., 2015b).

**Fig. 6:**
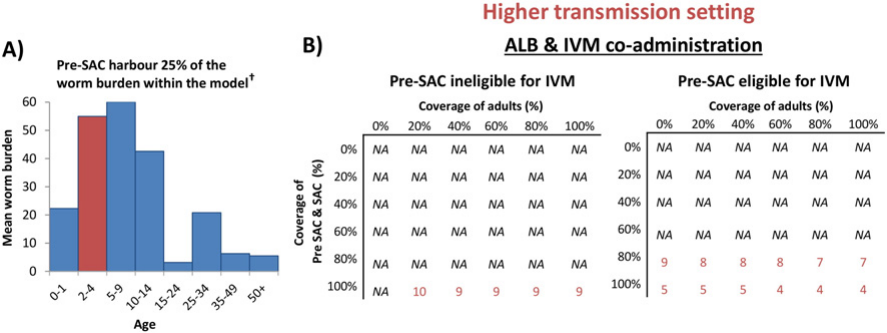
Mean intensity of infection in different age groupings (A) and the number of years of annual treatment to achieve elimination of *T. trichiura* with and without Pre-SAC being eligible for ivermectin co-administration (B). The age profile of infection intensity used to parameterise the model (Fig. 1) is taken from (Bundy et al., 1987). ^†^Accounting for both the age profile of infection intensity and the assumed demography (Truscott et al., 2014) yields an estimate of 25% of the worms occurring in Pre-SAC. Results in panel B) pertain to a fitted (higher) transmission setting (R_0_ = 1.75). Durations over ten years were not considered (marked as NA (not achievable)). ALB; Albendazole, IVM; Ivermectin.

In the higher transmission setting our projections indicated elimination may not be feasible with preventive chemotherapy alone (even with ivermectin co-administration) (Figs. 5 and 6)*.* This highlights the need for complementary control interventions such as water, sanitation and hygiene (WASH) (Pullan et al., 2014b) and improvements in health education.

### 4.3 The importance of preschool-aged children

Our projections indicate that if ivermectin or another alternative treatment could be given to Pre-SAC, heavy infections would be further reduced (Fig. 4 and Supporting Fig. S3) and the timeline to elimination would be shorter — at least in settings where Pre-SAC have a high proportion of the overall worm burden (Bundy et al., 1987) (Fig. 6). This analysis indicates that it would be advantageous for Pre-SAC to be eligible for any novel treatment strategy for *T. trichiura* (Albonico et al., 2008), particularly if programmatic goals shift towards breaking transmission (Fig. 6)*.*

### 4.4 Limitations

Due to the limited availability of data, there remains uncertainty surrounding the parameterisation of the key epidemiological processes of *T. trichiura* transmission models. This is particularly true for the strength of the density dependence acting on female worm fecundity — which requires worm expulsion data to parameterise.

**Fig. 7:**
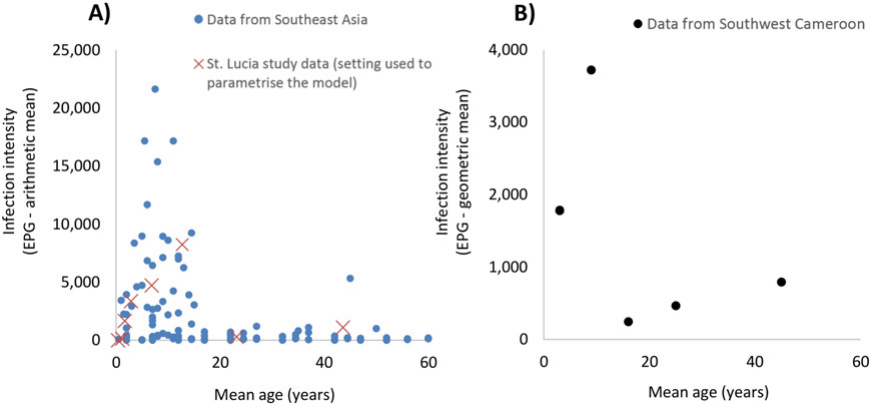
Observed age profiles of *T. trichiura* infection intensity in Southeast Asia (A) and Southwest Cameroon (B). Panel A contains EPG data from 11 studies (18 areas in four different countries) identified by a systematic review of epidemiological surveys of STH in Southeast Asia (Dunn et al., 2016) (compared to data from the St. Lucia study (Bundy et al., 1987)). Panel B reproduces that data provided by a study from Cameroon (Mbuh et al., 2012). EPG: eggs per gramme of faeces.

It is important to note that the projections regarding the added benefit of treating Pre-SAC and the limited impact of expanding mass treatment to include adults are highly dependent on the assumed age profile of infection intensity (Figs. 1 and 6) — which is used to parameterise the relative exposure of different age groups to eggs in the environment. Though, the absence of alternative age stratified intensity datasets from other settings remains an important limitation of this study, the assumed profile and lower burden in adults (Fig. 1) is consistent with the overall pattern found by a recent systematic review of epidemiological surveys of STH in Southeast Asia (Fig. 7A) (Dunn et al., 2016) and data from a study in Cameron (Mbuh et al., 2012) (Fig. 7B). That said there will be variations in this infection profile, and mass treatment of adults may be needed in some settings (furthermore pregnant women (a recognised risk group) may require treatment independent of this (Gyorkos et al., 2012)). More cross-sectional studies that measure both egg counts and worm burden are required to allow a range of different age profiles of infection intensity to be explored in the sensitivity analysis. It is important to note that converting EPG measurements to worm burden is not straightforward and would require a robust model of diagnostic sensitivity. This is an important area of future research for STH transmission models, as it will allow significantly more data to be used in model parameterization and validation.

Within the model, a worm burden above an age specific threshold was used as a proxy for disease (a standard method in the published literature (Chan et al., 1994, Turner et al., 2015a, Medley et al., 1993)). However, it is important to acknowledge that these thresholds (Table 1) are uncertain, and are likely to be influenced by a number of host specific factors such as nutritional status (Turner et al., 2016, Brooker, 2010). These results further highlight that the reduction in prevalence of infection is a poor effectiveness metric when evaluating the impact of preventive chemotherapy on morbidity (which is linked to heavy infections). Because the number of prevalent case years averted is often larger in lower transmission settings (Table 2) this metric would indicate it is more cost-effective to treat in lower compared to higher transmission settings (the opposite to what is observed when using metrics based on intensity (Turner et al., Epub ahead of print)).

It should be noted that uncertainty surrounds the precise treatment efficacies against *T. trichiura* — partly due to the limitations of the current widely used diagnostic tool of faecal egg examination (Nikolay et al., 2014). However, the relative increase in the treatment efficacy when co-administering ivermectin was parameterised based on randomized controlled trials (Knopp et al., 2010, Speich et al., 2015) and is supported by other studies (Beach et al., 1999, Belizario, 2003).

Furthermore, these projections do not account for any non-compliance to treatment and assume that everyone has an equal probability of taking each treatment. Consequently, the benefit of increasing the treatment frequency will probably be overestimated (as it does not account for individuals systematically missing both treatment rounds — which can reduce the benefit of increasing the treatment frequency (Turner et al., 2014a)).

### 4.5 Research needs

Ivermectin is currently donated for onchocerciasis and lymphatic filariasis control (25 Years: The MECTIZANⓇ Donation Program), but not directly for STH. Consequently, the actual financial cost of ivermectin and/or its potential economic value if it were to be donated for STH needs to be estimated to allow future cost-effectiveness analysis (an important gap for NTDs (Turner et al., 2014b, Turner et al., 2015a, Lee et al., 2015)).

It is important to consider that the use of ivermectin against *T. trichiura* will also have additional beneficial effects against Strongyloides infection (Datry et al., 1994), residual levels of onchocerciasis and lymphatic filariasis (Ottesen et al., 2008), and against co-endemic ectoparasites (such as scabies) (Dourmishev et al., 2005, Heukelbach et al., 2004).

Ivermectin co-administration is not the only potential solution for the limited efficacy of the standard treatments against *T. trichiura*, and there is ongoing research on other drugs/drug combinations (such as: triple dosing with standalone albendazole or mebendazole, pyrantel–oxantel (Albonico et al., 2002), oxantel pamoate–albendazole (Speich et al., 2015, Keiser et al., 2014) and papaya cysteine proteinases (Levecke et al., 2014b)). This analysis indicates it would be advantageous for Pre-SAC to be eligible for any new treatment combinations. Dynamic transmission models can be useful tools for comparing the impact of the different alternative drugs/combinations (not only for *T. trichiura*, but the other STH species as well). This will be important for gaining a comprehensive understanding of the impact of the different potential drugs on all of the STH.

An important programmatic consideration for treating continuously with drugs that do not effectively clear *T. trichiura* is the potential risk of drug resistance developing (Vercruysse et al., 2011b, Olliaro et al., 2011). The use of drug combinations may be an important method for delaying or avoiding drug resistance (as using drugs with different modes of action can make it harder for resistance to develop and spread). This aspect should be an important consideration in the evaluation of the various potential treatment alternatives for *T. trichiura* (Diawara et al., 2013). As STH programmes expand over the coming years, further research in methods for detecting resistance and controlling its spread is urgently needed (Vercruysse et al., 2011b, Barda et al., 2015).

A positive finding of this study is that current lymphatic filariasis treatment programmes, which include community-wide ivermectin–albendazole combination therapy, may be having a large impact on *T. trichiura*. This is supported by observations in the field (Massa et al., 2009). For this reason, future studies into the impact that scale-down or completion of LF control programmes will have on *T. trichiura* in co-endemic areas is required. This will become increasingly important as lymphatic filariasis programmes move towards elimination.

### 4.6 Conclusion

Although the current STH programmes providing child-targeted preventive chemotherapy with annual standalone albendazole or mebendazole reduce *T. trichiura* worm burdens, the impact for children is much lower compared to what can be seen for the other STH (Truscott et al., 2014, Truscott et al., 2015, Anderson et al., 2014). In addition, these simulations indicate that in high transmission settings the standard treatments may be insufficient to fully control prevalence of heavy infections. Co-administering ivermectin greatly increased the projected impact of the programme in these high transmission settings, particularly in settings where biannual treatment is not possible.

When using the current standalone treatments, breaking transmission was projected to be infeasible in most settings. Ivermectin co-administration greatly increased the feasibility of and timeframe for elimination. This has particularly important consequences if STH control programmes shift their goals to the interruption of transmission. However, the benefit of ivermectin co-administration is limited by the fact that Pre-SAC are mostly ineligible for ivermectin. It would advantageous for this age group to be made eligible for any novel treatment strategy against *T. trichiura*.

## Funding

HCT and AAB are supported by London Centre for Neglected Tropical Disease Research (funded by GSK). JET and TDH are supported by the Bill & Melinda Gates Foundation (#OPP1033751) and the Partnership for Child Development. SJB is supported by a Wellcome Trust Senior Fellowship in Basic Biomedical Science (098045). RMA is also supported by the Bill & Melinda Gates Foundation. The funders had no role in study design, data collection and analysis, decision to publish, or preparation of the manuscript.

## Conflict of interest

Roy M. Anderson is a non-executive member of the board of GlaxoSmithKline (GSK). GlaxoSmithKline played no role in study design, data collection and analysis, decision to publish, or preparation of the manuscript.

## Authors' contributions

HCT conducted the analysis and drafted the first version of the manuscript. JET coded the model and performed the parameter estimation. JET, AAB, TDH, SJB, and RMA contributed to the design of the study and writing of the paper. All authors read and approved the final version of the manuscript.

## Appendix A Supplementary data

Supplementary material Image 1

## References

[bb0005] 25 Years: The MECTIZAN® Donation Program [http://www.merck.com/about/featured-stories/mectizan1.html].

[bb0010] Albonico M., Bickle Q., Haji H.J., Ramsan M., Khatib K.J., Montresor A., Savioli L., Taylor M. (2002). Evaluation of the efficacy of pyrantel–oxantel for the treatment of soil-transmitted nematode infections. Trans. R. Soc. Trop. Med. Hyg..

[bb0015] Albonico M., Allen H., Chitsulo L., Engels D., Gabrielli A.-F., Savioli L. (2008). Controlling soil-transmitted helminthiasis in pre-school-age children through preventive chemotherapy. PLoS Negl. Trop. Dis..

[bb0020] Anderson R.M., May R.M. (1985). Helminth infections of humans: mathematical models, population dynamics, and control. Adv. Parasitol..

[bb0025] Anderson R.M., Truscott J.E., Hollingsworth T.D. (2014). The coverage and frequency of mass drug administration required to eliminate persistent transmission of soil-transmitted helminths. Philos. Trans. R. Soc. Lond. Ser. B Biol. Sci..

[bb0030] Anderson R.M., Turner H.C., Truscott J.E., Hollingsworth T.D., Brooker S.J. (2015). Should the goal for the treatment of soil transmitted helminth (STH) infections be changed from morbidity control in children to community-wide transmission elimination?. PLoS Negl. Trop. Dis..

[bb0035] Barda B.D., Keiser J., Albonico M. (2015). Human trichuriasis: diagnostics update. Curr. Trop. Med. Rep..

[bb0040] Beach M.J., Streit T.G., Addiss D.G., Prospere R., Roberts J.M., Lammie P.J. (1999). Assessment of combined ivermectin and albendazole for treatment of intestinal helminth and *Wuchereria bancrofti* infections in Haitian schoolchildren. Am.J.Trop. Med. Hyg..

[bb0045] Belizario V.Y., ME Amarillo, WU de Leon, AE de los Reyes, MG Bugayong, BJ Macatangay (2003). A comparison of the efficacy of single doses of albendazole, ivermectin, and diethylcarbamazine alone or in combinations against *Ascaris* and *Trichuris* spp. Bull. World Health Organ..

[bb0100] Bill & Melinda Gates Foundation (2014). Global Partners Are Taking the “Neglect” out of “Neglected Tropical Diseases”. http://www.gatesfoundation.org/Media-Center/Press-Releases/2014/04/Global-Partners-Are-Taking-the-Neglect-out-of-Neglected-Tropical-Diseases.

[bb0050] Brooker S. (2010). Estimating the global distribution and disease burden of intestinal nematode infections: adding up the numbers — a review. Int. J. Parasitol..

[bb0055] Brooker S.J., Nikolay B., Balabanova D., Pullan R.L. (2015). Global feasibility assessment of interrupting the transmission of soil-transmitted helminths: a statistical modelling study. Lancet Infect. Dis.

[bb0060] Bundy D.A., Cooper E.S. (1989). *Trichuris* and trichuriasis in humans. Adv. Parasitol..

[bb0065] Bundy D.A., Cooper E.S., Thompson D.E., Anderson R.M., Didier J.M. (1987). Age-related prevalence and intensity of *Trichuris trichiura* infection in a St. Lucian community. Trans. R. Soc. Trop. Med. Hyg..

[bb0070] Bundy D., Chan M., Medley G., Jamison D., Savioli L., Murray C.J.L., Lopez A.D., Mathers C.D. (2004). Intestinal nematode infections. Global Epidemiology of Infectious Disease.

[bb0075] Chan M.S., Medley G.F., Jamison D., Bundy D.A. (1994). The evaluation of potential global morbidity attributable to intestinal nematode infections. Parasitology.

[bb0080] Datry A., Hilmarsdottir I., Mayorga-Sagastume R., Lyagoubi M., Gaxotte P., Biligui S., Chodakewitz J., Neu D., Danis M., Gentilini M. (1994). Treatment of *Strongyloides stercoralis* infection with ivermectin compared with albendazole: results of an open study of 60 cases. Trans. R. Soc. Trop. Med. Hyg..

[bb0085] Diawara A., Halpenny C.M., Churcher T.S., Mwandawiro C., Kihara J., Kaplan R.M., Streit T.G., Idaghdour Y., Scott M.E., Basáñez M.-G. (2013). Association between response to albendazole treatment and β-tubulin genotype frequencies in soil-transmitted helminths. PLoS Negl. Trop. Dis..

[bb0090] Dourmishev A.L., Dourmishev L.A., Schwartz R.A. (2005). Ivermectin: pharmacology and application in dermatology. Int. J. Dermatol..

[bb0095] Dunn J.C., Turner H.C., Tun A., Anderson R.M. (2016). Epidemiological surveys of, and research on, soil-transmitted helminths in Southeast Asia: a systematic review. Parasite Vectors.

[bb0105] Goldman A.S., Guisinger V.H., Aikins M., Amarillo M.L., Belizario V.Y., Garshong B., Gyapong J., Kabali C., Kamal H.A., Kanjilal S. (2007). National mass drug administration costs for lymphatic filariasis elimination. PLoS Negl. Trop. Dis..

[bb0110] Gyorkos T.W., Gilbert N.L., Larocque R., Casapía M., Montresor A. (2012). Re-visiting *Trichuris trichiura* intensity thresholds based on anemia during pregnancy. PLoS Negl. Trop. Dis..

[bb0115] Heukelbach J., Winter B., Wilcke T., Muehlen M., Albrecht S., de Oliveira F.A., Kerr-Pontes L.R., Liesenfeld O., Feldmeier H. (2004). Selective mass treatment with ivermectin to control intestinal helminthiases and parasitic skin diseases in a severely affected population. Bull. World Health Organ..

[bb0120] Keiser J., Utzinger J. (2008). Efficacy of current drugs against soil-transmitted helminth infections: systematic review and meta-analysis. JAMA.

[bb0125] Keiser J., Speich B., Utzinger J. (2014). Oxantel pamoate–albendazole for *Trichuris trichiura* infection. N. Engl. J. Med..

[bb0130] Knopp S., Mohammed K.A., Speich B., Hattendorf J., Khamis I.S., Khamis A.N., Stothard J.R., Rollinson D., Marti H., Utzinger J. (2010). Albendazole and mebendazole administered alone or in combination with ivermectin against *Trichuris trichiura*: a randomized controlled trial. Clin. Infect. Dis..

[bb0135] Lee B.Y., Bartsch S.M., Gorham K.M. (2015). Chapter eight — economic and financial evaluation of neglected tropical diseases. Advances in Parasitology.

[bb0140] Levecke B., Montresor A., Albonico M., Ame S.M., Behnke J.M., Bethony J.M., Noumedem C.D., Engels D., Guillard B., Kotze A.C. (2014). Assessment of anthelmintic efficacy of mebendazole in school children in six countries where soil-transmitted helminths are endemic. PLoS Negl. Trop. Dis..

[bb0145] Levecke B., Buttle D.J., Behnke J.M., Duce I.R., Vercruysse J. (2014). Cysteine proteinases from papaya (*Carica papaya*) in the treatment of experimental *Trichuris suis* infection in pigs: two randomized controlled trials. Parasite Vectors.

[bb0150] Massa K., Magnussen P., Sheshe A., Ntakamulenga R., Ndawi B., Olsen A. (2009). The combined effect of the lymphatic filariasis elimination programme and the schistosomiasis and soil-transmitted helminthiasis control programme on soil-transmitted helminthiasis in schoolchildren in Tanzania. Trans. R. Soc. Trop. Med. Hyg..

[bb0155] Mbuh J.V., Ntonifor N.H., Ojong J. (2012). The epidemiology of soil-transmitted helminth and protozoan infections in south-west Cameroon. J. Helminthol..

[bb0160] Medley G.F., Guyatt H.L., Bundy D.A. (1993). A quantitative framework for evaluating the effect of community treatment on the morbidity due to ascariasis. Parasitology.

[bb0165] Nikolay B., Brooker S.J., Pullan R.L. (2014). Sensitivity of diagnostic tests for human soil-transmitted helminth infections: a meta-analysis in the absence of a true gold standard. Int. J. Parasitol..

[bb0170] Olliaro P., Seiler J., Kuesel A., Horton J., Clark J.N., Don R., Keiser J. (2011). Potential drug development candidates for human soil-transmitted helminthiases. PLoS Negl. Trop. Dis..

[bb0175] Ottesen E.A., Hooper P.J., Bradley M., Biswas G. (2008). The global programme to eliminate lymphatic filariasis: health impact after 8 years. PLoS Negl. Trop. Dis..

[bb0180] Pullan R.L., Brooker S.J. (2012). The global limits and population at risk of soil-transmitted helminth infections in 2010. Parasite Vectors.

[bb0185] Pullan R.L., Smith J.L., Jasrasaria R., Brooker S.J. (2014). Global numbers of infection and disease burden of soil transmitted helminth infections in 2010. Parasite Vectors.

[bb0190] Pullan R.L., Freeman M.C., Gething P.W., Brooker S.J. (2014). Geographical inequalities in use of improved drinking water supply and sanitation across Sub-Saharan Africa: mapping and spatial analysis of cross-sectional survey data. PLoS Med..

[bb0195] Speich B., Ali S.M., Ame S.M., Bogoch I.I., Alles R., Huwyler J., Albonico M., Hattendorf J., Utzinger J., Keiser J. (2015). Efficacy and safety of albendazole plus ivermectin, albendazole plus mebendazole, albendazole plus oxantel pamoate, and mebendazole alone against *Trichuris trichiura* and concomitant soil-transmitted helminth infections: a four-arm, randomised controlled trial. Lancet Infect. Dis..

[bb0200] Truscott J.E., Hollingsworth T.D., Brooker S.J., Anderson R.M. (2014). Can chemotherapy alone eliminate the transmission of soil transmitted helminths?. Parasit Vectors.

[bb0205] Truscott J.E., Turner H.C., Anderson R.M. (2015). What impact will the achievement of the current World Health Organisation targets for anthelmintic treatment coverage in children have on the intensity of soil transmitted helminth infections?. Parasites Vectors.

[bb0210] Turner H.C., Osei-Atweneboana M.Y., Walker M., Tettevi E.J., Churcher T.S., Asiedu O., Biritwum N.-K., Basáñez M.G. (2013). The cost of annual versus biannual community-directed treatment with ivermectin: Ghana as a case study. PLoS Negl. Trop. Dis..

[bb0215] Turner H.C., Walker M., Churcher T.S., Osei-Atweneboana M.Y., Biritwum N.-K., Hopkins A., Prichard R.K., Basáñez M.-G. (2014). Reaching the London declaration on neglected tropical diseases goals for onchocerciasis: an economic evaluation of increasing the frequency of ivermectin treatment in Africa. Clin. Infect. Dis.

[bb0220] Turner H.C., Walker M., French M.D., Blake I.M., Churcher T.S., Basáñez M.G. (2014). Neglected tools for neglected diseases: mathematical models in economic evaluations. Trends Parasitol..

[bb0225] Turner H.C., Truscott J.E., Hollingsworth T.D., Bettis A.A., Brooker S.J., Anderson R.M. (2015). Cost and cost-effectiveness of soil-transmitted helminth treatment programmes: systematic review and research needs. Parasite Vectors.

[bb0230] Turner H.C., Truscott J.E., Bettis A.A., Shuford K.V., Dunn J.C., Hollingsworth T.D., Brooker S.J., Anderson R.M. (2015). An economic evaluation of expanding hookworm control strategies to target the whole community. Parasite Vectors.

[bb0235] Turner H.C., Truscott J.E., Fleming F.M., Hollingsworth T.D., Brooker S.J., Anderson R.M. (2016). Cost-effectiveness of scaling up mass drug administration for the control of soil-transmitted helminths: a comparison of cost function and constant costs analyses. Lancet Infect. Dis..

[bb0240] Vercruysse J., Behnke J.M., Albonico M., Ame S.M., Angebault C., Bethony J.M., Engels D., Guillard B., Nguyen T.V., Kang G. (2011). Assessment of the anthelmintic efficacy of albendazole in school children in seven countries where soil-transmitted helminths are endemic. PLoS Negl. Trop. Dis..

[bb0245] Vercruysse J., Albonico M., Behnke J.M., Kotze A.C., Prichard R.K., McCarthy J.S., Montresor A., Levecke B. (2011). Is anthelmintic resistance a concern for the control of human soil-transmitted helminths?. Int. J. Parasitol..

[bb0250] World Health Organization (2006). Preventive Chemotherapy in Human Helminthiasis: Coordinated Use of Anthelminthic Drugs in Control Interventions: a Manual for Health Professionals and Programme Managers.

[bb0255] World Health Organization (2012). Eliminating soil-transmitted helminthiasis as a public health problem in children: progress report 2001–2010 and strategic plan 2011–2020.

[bb0260] World Health Organization (2012). Accelerating work to overcome the global impact of neglected tropical diseases– A roadmap for implementation. http://www.who.int/neglected_diseases/NTD_RoadMap_2012_Fullversion.pdf.

[bb0265] Yap P., Wu F.W., Du Z.W., Hattendorf J., Chen R., Jiang J.Y., Kriemler S., Krauth S.J., Zhou X.N., Utzinger J. (2014). Effect of deworming on physical fitness of school-aged children in Yunnan, China: a double-blind, randomized, placebo-controlled trial. PLoS Negl. Trop. Dis..

