# Supplementary material for: Analysis of the population-level impact of co-administering ivermectin with albendazole or mebendazole for the control and elimination of Trichuris trichiura
Source: Parasite Epidemiol Control. 2016 Mar 2;1(2):177–87. doi: 10.1016/j.parepi.2016.02.004 (PMC4946157; doi:10.1016/j.parepi.2016.02.004)
Supplement: Supplementary file 1 — Supplementary material [file mmc1.pdf]

Supporting information

The Model

The fundamental model used to describe the mean worm burden of individuals of a given age and the quantity of infectious eggs in the environment is taken from Anderson and May [1]. The current version of the model is described in detail in Truscott *et al* and Anderson *et al* [2-4].

For the purposes of this analysis the models output, which is in terms of the mean number of female worms [2, 3], was converted to the mean number of worms (both males and females) assuming a 1:1 sex ratio [5, 6] i.e. the modelled female worm burden output was doubled.

In this paper we used the model parameters pertaining to *Trichuris trichiura*, described in Table S1.

Supporting Table S1: Model parameters for *Trichuris trichiura*

Parameter	Value	Source
Adult worm life expectancy (years)	1	[1, 7]
Density dependent fecundity, γ	0.0035	Fitted to the data from [7]
$z=e^{-\gamma}$	0.9965	-
Aggregation parameter, k	0.38	Fitted to the data from [7]
Life expectancy of the infective stage	30 days	[1]
Relative values for the degree of exposure, β, and contribution, ρ, of the various age groups to the infectious reservoir	0-2 year olds = 0.5,<br>3-7 year olds = 2.13,<br>8-12 year olds = 1,<br>12< year olds = 0.28	Fitted to the data from [7]
Drug efficacy (proportion of worms expelled per treatment)	Varied	See Methods
Proportion of humans at age a, $P(a)$	Based on Uganda's demographical profile	[3, 8]

Fitting

Model parameters were estimated by fitting to data from the cross-sectional study of *T. trichiura* conducted in St. Lucia by Bundy e*t al* [7]. The study collected pre-treatment and post-treatment stool samples from 119 individuals across a full range of ages. The resulting data set contains a record for each individual consisting of age, eggs per gram (EPG) of faeces and expelled worm burden. We assume that the study population has not been subject to any recent chemotherapeutic interventions and that therefore the age profile data can be assumed to be at equilibrium.

Fitting was achieved by using appropriate probabilistic models or sub-models to generate a likelihood for the data. Then Bayesian techniques can be used to construct a posterior distribution for the parameter values, from which can be drawn maximum likelihood estimators for their values.

Density dependent fecundity, γ. The density-dependent fecundity parameter, γ, can be estimated from data that links the number of female worms in an individual, w, with their egg output, E, in eggs per gram, for example. Within our current model, the mean egg output for individual is described by,

$$\bar{E} = \lambda w \exp(-\gamma w)$$

We assume a negative binomial (NB) as the distribution of individual EPG values between samples. This form of over-dispersion has been observed for a number of helminth species [9-11]. The likelihood expression for paired worm/EPG data is,

$$L(\lambda, \gamma, k_{epg}) = \prod_{i}^{N} NB(E_i; n_i, \lambda, \gamma, k_{epg})$$

where k_{epg} is the aggregation parameter for the negative binomial distribution. Given non-informative priors, the maximum likelihood estimator for γ is 0.0035. Estimators for λ and k_{epg} are 140 EPG/worm and 0.82, respectively, but these parameters are absorbed into other parameter groupings within the model as used in this context.

Our model assumes that the distribution of worms among hosts is aggregated according to negative binomial distribution. Fitting a negative binomial distribution to the individual female worm burdens gives a best estimate for the aggregation parameter, k, of 0.38. This estimate may be somewhat biased from the population-level value as the age distribution of the sample population is not identical to that of the population as a whole, as shown in [7].

Our deterministic model (described in the first paragraph) follows the evolution of the mean worm burden in a given age category, *M(a)*. The model can be interpreted as a probabilistic model by recalling that the probability of any given worm burden, n, in an individual of age, a, will be distributed according to a negative binomial distribution, $n \sim NB(M(a), k)$.

Hence for paired age/worm data $\{a_i, n_i\}$ for individual *i*, the likelihood is given by,

$$L(\Theta) = \prod_{i}^{N} NB(n_i; M(a_i), \Theta)$$

where Θ represents a vector of parameters, including age dependent infectious contact rate for hosts, *β(a)*, and R_0. MLE values are used for parameters whose fitting has already been described [4, 12]. Strictly, we should use the posteriors from the previous fittings as the priors for the current likelihood, but in practice this doesn't have a significant impact on the final values.

Supporting Table S2: Sensitivity of the relative increase in effects gained by co-administering ivermectin with albendazole to the treatment coverage (in comparison with targeted albendazole monotherapy).

Treatment coverage	Worm years averted	Prevalent case years averted	Heavy infection case years averted – lower threshold	Heavy infection case years averted – higher threshold
85%	41%	107%	42%	20%
75%	39%	126%	36%	15%
65%	35%	152%	29%	10%

Results pertain to the fitted (higher) transmission setting (R_0=1.75) and a targeted preventive chemotherapy programme treating Pre-SAC and SAC. The analysis was performed with a ten year time horizon (comparing ten years of standalone treatment to ivermectin co-administration). Note that those under five years of age did not receive ivermectin and would only be treated with albendazole. The thresholds for heavy infection are presented in Table 1.

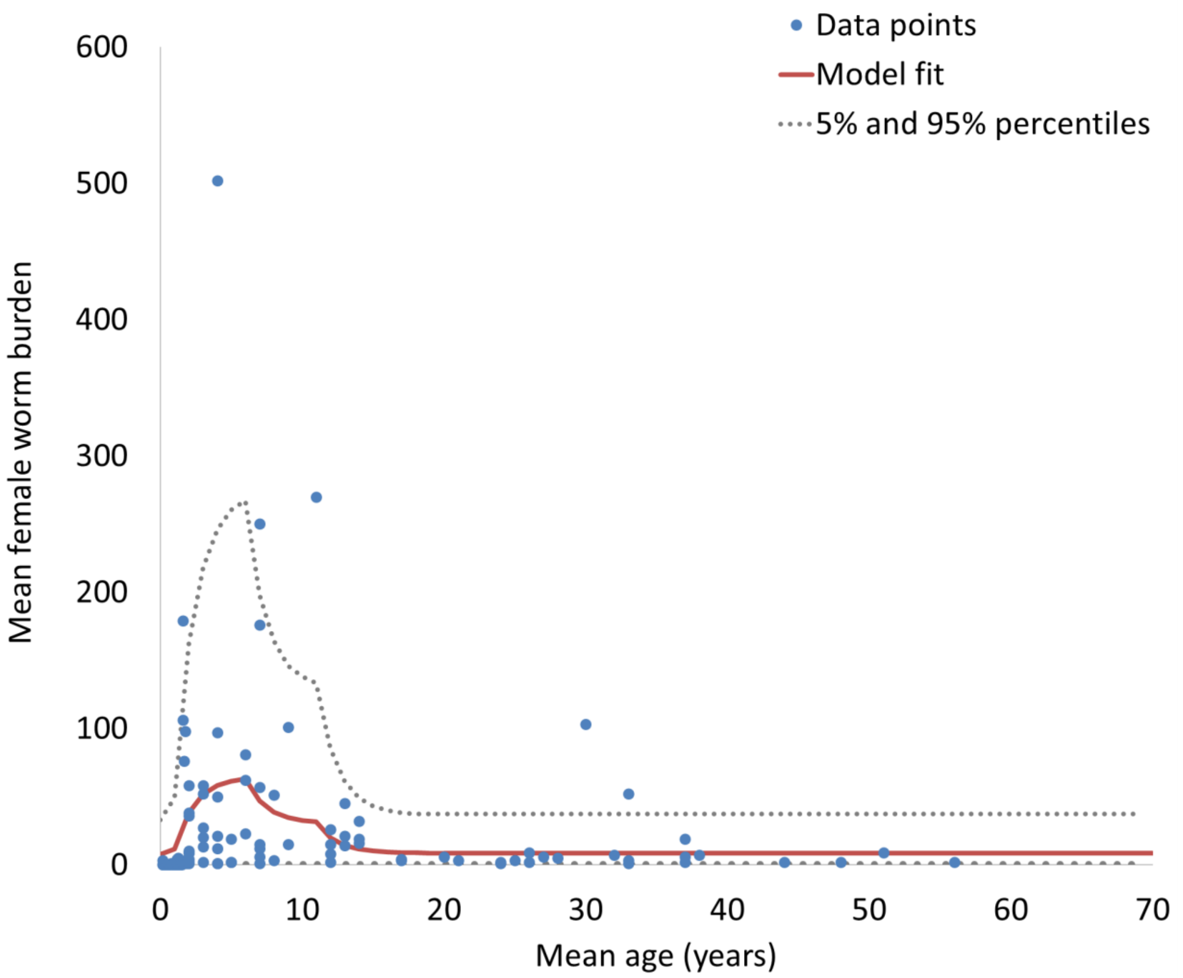

Supporting Figure S1: Model fit to cross-sectional data from the St. Lucia study [7]. *Individual data points for female worm burden are represented by crosses. The solid line is equilibrium mean worm burden with age given by the model with the broken lines indicating the 5th and 95th percentile for the underlying negative binomial distribution. Note that Figure 1 of the main text is showing total (and not female) worm burden.*

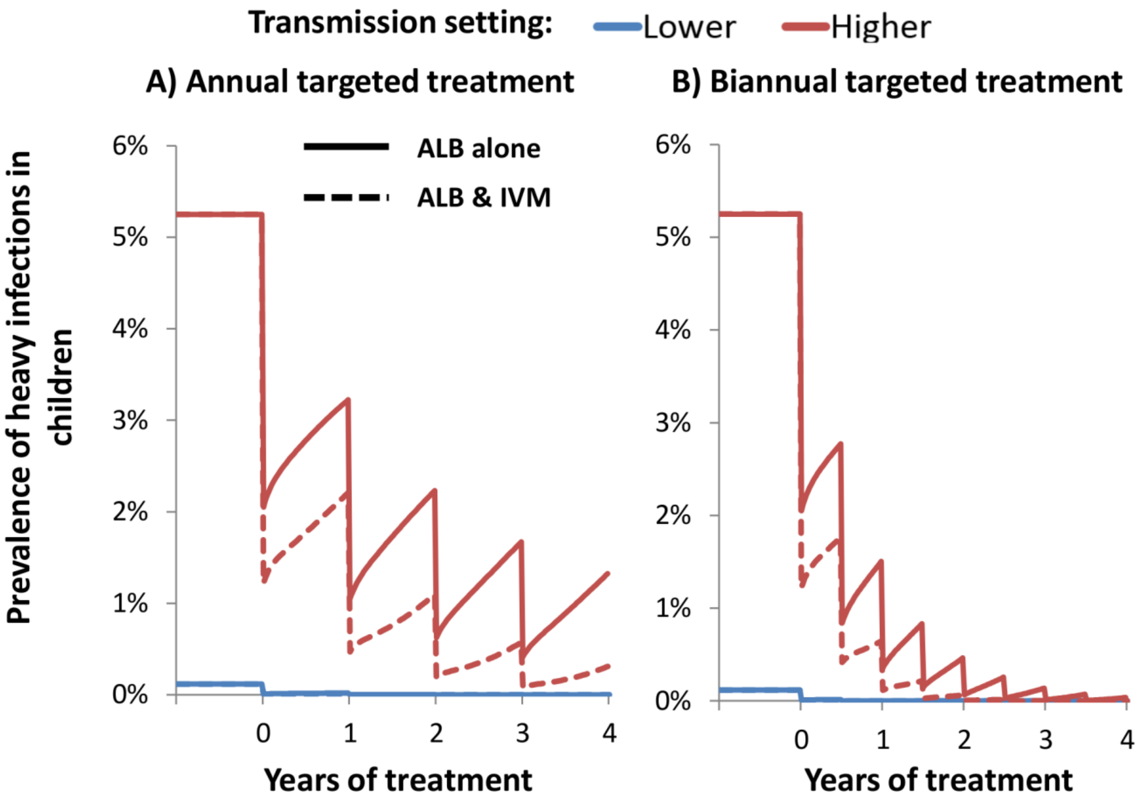

Supporting Figure S2: Projected impact of annual and biannual child-targeted preventive chemotherapy with and without ivermectin co-administration on the prevalence of heavy *T. trichiura* infections in children. *The results assume the higher intensity thresholds for heavy infection (presented in Table 1 of the main text). The solid and dotted lines pertain to standalone albendazole, and albendazole-ivermectin co-administration respectively. Two different transmission settings were explored; lower (R_0=1.25), and higher (R_0=1.75 – fitted). Results assume 75% treatment coverage of Pre-SAC and SAC. The drug efficacy was assumed to be 50% for standalone albendazole [13], and 95% when co-administering ivermectin [14]. Note that those under five years of age cannot receive ivermectin and would only be treated with albendazole. ALB; Albendazole, IVM; Ivermectin.*

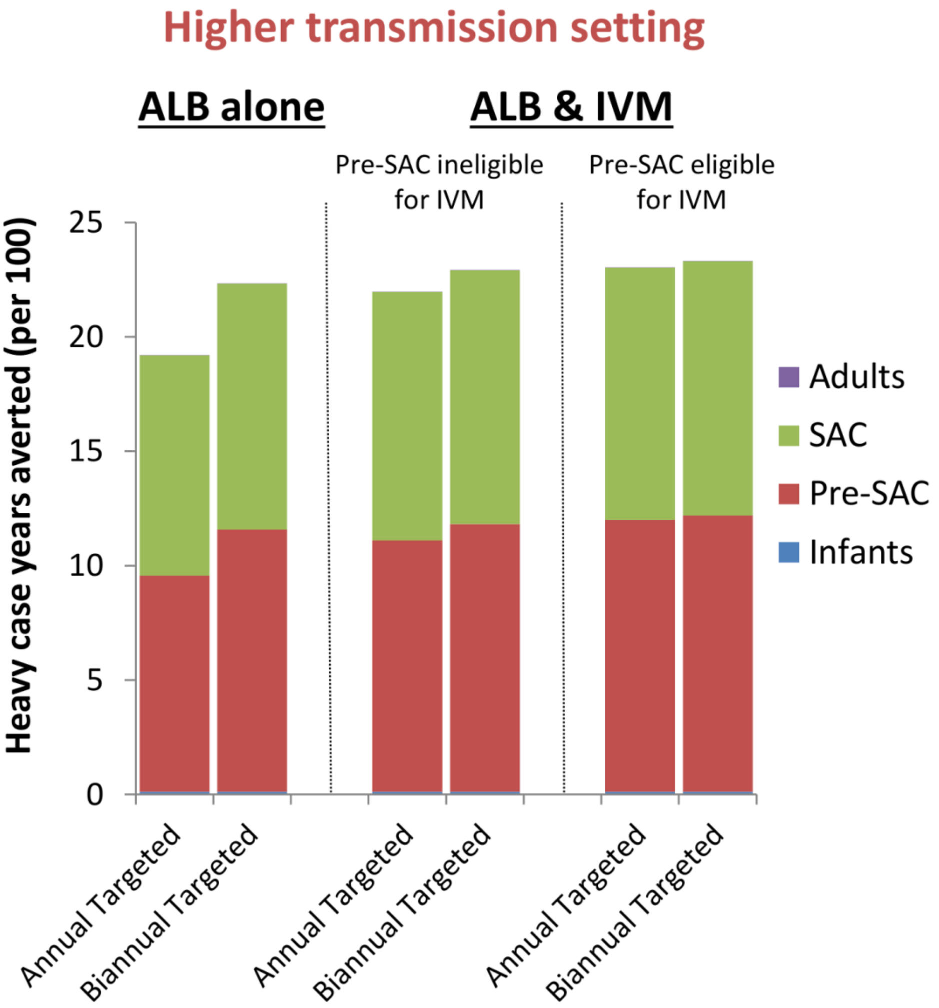

Supporting Figure S3: Impact of a child-targeted preventive chemotherapy in terms of heavy case years averted with and without ivermectin co-administration. *The results assume the higher intensity thresholds for heavy infection (presented in Table 1). The bars are stratified by the host age-group (from the bottom up: infants, Pre-SAC, SAC and adults). Results pertain to the fitted (higher) transmission setting (R_0=1.75) and assume 75% treatment coverage of Pre-SAC and SAC. The analysis was performed with a ten year time horizon (comparing ten years of standalone treatment to IVE co-administration). The drug efficacy was assumed to be 50% for standalone albendazole* [13], *and 95% when co-administering ivermectin* [14]. *ALB; Albendazole, IVM; Ivermectin.*

Higher transmission setting

MBZ alone

Coverage of Pre-SAC & SAC (%) \ Coverage of adults (%)	0%	20%	40%	60%	80%	100%
0%	*NA*	*NA*	*NA*	*NA*	*NA*	*NA*
20%	*NA*	*NA*	*NA*	*NA*	*NA*	*NA*
40%	*NA*	*NA*	*NA*	*NA*	*NA*	*NA*
60%	*NA*	*NA*	*NA*	*NA*	*NA*	*NA*
80%	*NA*	*NA*	*NA*	*NA*	*NA*	*NA*
100%	*NA*	*NA*	*NA*	*NA*	*NA*	*NA*

MBZ & IVM co-administration

Pre-SAC ineligible for IVM

Coverage of Pre-SAC & SAC (%) \ Coverage of adults (%)	0%	20%	40%	60%	80%	100%
0%	*NA*	*NA*	*NA*	*NA*	*NA*	*NA*
20%	*NA*	*NA*	*NA*	*NA*	*NA*	*NA*
40%	*NA*	*NA*	*NA*	*NA*	*NA*	*NA*
60%	*NA*	*NA*	*NA*	*NA*	*NA*	*NA*
80%	*NA*	*NA*	*NA*	*NA*	*NA*	*NA*
100%	8	8	7	7	7	7

Pre-SAC eligible for IVM

Coverage of Pre-SAC & SAC (%) \ Coverage of adults (%)	0%	20%	40%	60%	80%	100%
0%	*NA*	*NA*	*NA*	*NA*	*NA*	*NA*
20%	*NA*	*NA*	*NA*	*NA*	*NA*	*NA*
40%	*NA*	*NA*	*NA*	*NA*	*NA*	*NA*
60%	*NA*	*NA*	*NA*	*NA*	*NA*	*NA*
80%	8	8	7	7	7	7
100%	5	5	4	4	4	4

Lower transmission setting

MBZ alone

Coverage of Pre-SAC & SAC (%) \ Coverage of adults (%)	0%	20%	40%	60%	80%	100%
0%	*NA*	*NA*	*NA*	*NA*	*NA*	*NA*
20%	*NA*	*NA*	*NA*	*NA*	*NA*	*NA*
40%	*NA*	*NA*	*NA*	*NA*	*NA*	*NA*
60%	8	7	7	7	7	7
80%	5	5	5	5	5	5
100%	4	4	4	4	4	4

MBZ & IVM co-administration

Pre-SAC ineligible for IVM

Coverage of Pre-SAC & SAC (%) \ Coverage of adults (%)	0%	20%	40%	60%	80%	100%
0%	*NA*	*NA*	*NA*	*NA*	*NA*	*NA*
20%	*NA*	*NA*	*NA*	*NA*	*NA*	*NA*
40%	9	9	9	8	8	8
60%	6	5	5	5	5	5
80%	4	4	4	4	3	3
100%	3	3	3	3	3	2

Pre-SAC eligible for IVM

Coverage of Pre-SAC & SAC (%) \ Coverage of adults (%)	0%	20%	40%	60%	80%	100%
0%	*NA*	*NA*	*NA*	*NA*	*NA*	*NA*
20%	*NA*	*NA*	*NA*	*NA*	*NA*	*NA*
40%	7	7	7	7	7	6
60%	4	4	4	4	4	4
80%	3	3	3	3	3	3
100%	2	2	2	2	2	2

Supporting Figure S4: Number of years-of annual treatment to achieve elimination of *T. trichiura* as a function of coverage of children versus adults. *Two different transmission settings were explored; lower (R_0=1.25), and higher (R_0=1.75 – fitted). Note that those under five years of age did not receive ivermectin and would only be treated with mebendazole. IVM; Ivermectin, MBZ; mebendazole. The corresponding results for albendazole as shown in Figure 5. Durations over ten years were not considered (marked as NA (not achievable)).*

References

1. Anderson RM, May RM: **Helminth infections of humans: mathematical models, population dynamics, and control**. *Adv Parasitol* 1985, **24**:1-101.
2. Truscott JE, Hollingsworth TD, Brooker SJ, Anderson RM: **Can chemotherapy alone eliminate the transmission of soil transmitted helminths?** *Parasit Vectors* 2014, **7**(1):266.
3. Anderson RM, Truscott JE, Hollingsworth TD: **The coverage and frequency of mass drug administration required to eliminate persistent transmission of soil-transmitted helminths**. *Philos Trans R Soc Lond B Biol Sci* 2014, **369**(1645):20130435.
4. Truscott JE, Turner HC, Anderson RM: **What impact will the achievement of the current World Health Organisation targets for anthelmintic treatment coverage in children have on the intensity of soil transmitted helminth infections?** *Parasites & Vectors* 2015, **8**(1):551.
5. Anderson RM: **The population dynamics and control of hookworm and roundworm infections**. In: *The Population Dynamics of Infectious Diseases: Theory and Applications.* Edited by Anderson RM: Springer US; 1982: 67-108.
6. Guyatt HL, Bundy DA: **Estimation of intestinal nematode prevalence: influence of parasite mating patterns**. *Parasitology* 1993, **107 (Pt 1)**:99-105.
7. Bundy DA, Cooper ES, Thompson DE, Anderson RM, Didier JM: **Age-related prevalence and intensity of *Trichuris trichiura* infection in a St. Lucian community**. *Trans R Soc Trop Med Hyg* 1987, **81**(1):85-94.
8. Pullan RL, Kabatereine NB, Quinnell RJ, Brooker S: **Spatial and genetic epidemiology of hookworm in a rural community in Uganda**. *PLoS Negl Trop Dis* 2010, **4**(6):e713.
9. McCullough FS, Bradley DJ: **Egg output stability and the epidemiology of Schistosoma haematobium. I. Variation and stability in Schistosoma haematobium egg counts**. *Trans R Soc Trop Med Hyg* 1973, **67**(4):475-490.
10. Anderson RM, Schad GA: **Hookworm burdens and faecal egg counts: an analysis of the biological basis of variation**. *Trans R Soc Trop Med Hyg* 1985, **79**(6):812-825.
11. Croll NA, Anderson RM, Gyorkos TW, Ghadirian E: **The population biology and control of Ascaris lumbricoides in a rural community in Iran**. *Trans R Soc Trop Med Hyg* 1982, **76**(2):187-197.
12. Truscott JE, Turner HC, Farrell SH, Anderson RM: **Soil Transmitted Helminths: mathematical models of transmission, the impact of mass drug administration and transmission elimination criteria**.
13. Vercruysse J, Behnke JM, Albonico M, Ame SM, Angebault C, Bethony JM, Engels D, Guillard B, Nguyen TV, Kang G *et al*: **Assessment of the anthelmintic efficacy of albendazole in school children in seven countries where soil-transmitted helminths are endemic**. *PLoS Negl Trop Dis* 2011, **5**(3):e948.
14. Speich B, Ali SM, Ame SM, Bogoch II, Alles R, Huwyler J, Albonico M, Hattendorf J, Utzinger J, Keiser J: **Efficacy and safety of albendazole plus ivermectin, albendazole plus mebendazole, albendazole plus oxantel pamoate, and mebendazole alone against Trichuris trichiura and concomitant soil-transmitted helminth infections: a four-arm, randomised controlled trial**. *Lancet Infect Dis* 2015, **15**(3):277-284.
